# Corruption in healthcare: global perspectives and the recent escalation of violence in Ecuador's public medicine procurement system

**DOI:** 10.3389/fpubh.2023.1259124

**Published:** 2023-11-08

**Authors:** Esteban Ortiz-Prado, Juan S. Izquierdo-Condoy, Jorge Vasconez-Gonzalez

**Affiliations:** One Health Research Group, Universidad de Las Américas, Quito, Ecuador

**Keywords:** corruption, violence, healthcare services, public health, low income

Corruption is a multifaceted phenomenon that involves dishonesty and criminal behavior perpetrated by individuals or organizations holding positions of authority. It encompasses the pursuit of unauthorized advantages and the exploitation of power for personal gain. This comprehensive definition, as outlined by UNODC's Action against Corruption and Economic Crime reflects a complex social, political, and economic issue with far-reaching implications for all nations (1, 2).

Corrupt practices have a significant impact in developing jurisdictions, such as Ecuador, as they undermine the integrity of institutions, hinder development, and harm society. In Ecuador, corruption can manifest itself in various forms, including bribery, embezzlement, and nepotism. The consequences of corruption are difficult to calculate and result in the erosion of public confidence in government and institutions. Corrupt practices distort the allocation of resources, diverting funds intended for essential public services such as healthcare, provision of medicines, education, and infrastructure (3). This disproportionately affects vulnerable populations, perpetuating inequality and hindering socioeconomic progress (4). Corrupt practices can severely compromise the justice system, preventing impartial trials and diminishing the rule of law. This environment can promote a culture where powerful figures avoid accountability. In the realm of healthcare corruption, several forms prevail. These encompass absenteeism, characterized by the persistent absence of healthcare professionals; informal payments, which are unrecorded contributions made by patients or their families in cash or kind; fraud, committed by various stakeholders such as providers, government inspectors, regulators, or payers; and finally, the misallocation of resources and pilferage of supplies (5).

This landscape becomes particularly complex in countries like Ecuador. The nation's healthcare system operates within a multifaceted and segmented structure, comprising multiple entities serving diverse population sectors. The Ministry of Public Health (Ministerio de Salud Pública), or MSP, is responsible for the healthcare of a vast number of citizens. Furthermore, specialized social security institutes like the Ecuadorian Social Security Institute (IESS) and those dedicated to police and army personnel (ISFFA and ISSPOL) also play crucial roles in healthcare delivery. Alongside these government-driven efforts, a significant portion of healthcare financing stems from out-of-pocket payments, mirroring trends seen in other Latin American nations (6).

In this context, Ecuador places significant emphasis on primary healthcare as the foundation of its healthcare system, focusing on community-based and preventive measures to enhance overall health outcomes and alleviate the demand for more specialized services (7). The country also acknowledges the significance of traditional and indigenous medicine, and efforts have been made to integrate these practices into the formal healthcare system, ensuring their safety and effectiveness. Moreover, out-of-pocket payments play a role in financing healthcare for a specific population segment (8). Additionally, a public-private partnership model is present, with the public sector aiming to provide healthcare services to all citizens and the private sector contributing to associated services and private healthcare. This partnership aims to ensure comprehensive healthcare coverage across the board. The National Agency for Regulation, Control, and Health Surveillance (ARCSA) holds the responsibility for regulating and supervising the quality, safety, and efficacy of drugs and pharmaceuticals. This pivotal role contributes to the maintenance of quality standards within the healthcare system (9).

The global healthcare landscape is increasingly tarnished by pervasive corruption, with severe repercussions on the quality and accessibility of health services, a situation acutely highlighted in developing nations. This widespread problem has been laid bare during the COVID-19 pandemic, where inflated prices, pervasive corrupt practices, and the proliferation of a black market for drugs and medical supplies have been starkly evident (10–12).

Globally, corruption in healthcare systems has far-reaching effects, extending beyond inflated costs to impact the availability and quality of essential drugs. The heavily regulated, complex, and often opaque nature of health systems provides ample opportunities for corruption to flourish at every point in the drug supply chain (10).

Pharmaceuticals, representing a significant portion of public health budgets, stand as the second-largest health sector expenditure following salaries, particularly pronounced in developing nations like Ecuador. In the context of Ecuador, the annual expenditure on medicine surpasses 1–1.5 billion dollars, with an overall spending of almost two billion dollars, predominantly within the public health sector (13). Despite this significant financial investment, the World Health Organization highlights a sobering fact: up to 50% of populations in low-income countries lack reliable access to quality essential medicines (14). This glaring deficiency finds its roots in the infiltration of fraud and corruption within the system, impeding access to safe and affordable medication. Notably, the countries most impacted by pandemics such as AIDS paradoxically stand as the most susceptible to corruption, owing to their fragile governance structures and lack of transparency (15).

The global healthcare landscape grapples with a pervasive challenge: corruption, fostered by inadequate government regulation, unrelenting bureaucratic pressures, and specific cultural contexts. This issue takes center stage, particularly pronounced in Latin American nations where a concerning inverse relationship exists between medication quality and corruption level (16).

Transparency and public accountability emerge as critical safeguards against corruption and key drivers of citizen engagement within public administrations. Comprehensive research spanning healthcare centers across Chile, Colombia, Ecuador, and Spain reveals a compelling nexus between transparency and factors like healthcare system architecture, internet accessibility, and administrative hierarchy. This interplay underscores the indispensable role of State participation in fostering information accessibility and nurturing a culture of social responsibility (17).

In Ecuador, corruption has become a critical issue within public health services, primarily witnessed in the procurement of medicines and medical supplies (18). Unethical practices such as collusion within the reverse auction system, bribery, price manipulation, procurement fraud, embezzlement, and nepotism have insidiously entrenched themselves into the very fabric of our healthcare system (19–21). However, the problem transcends these practices; it has been found that political mafias and drug lords may be involved in these corruption schemes. Infamously known cases, such as the Israeli citizen murdered in prison and another individual killed during a prison mob incident after publicly selling biosimilars procured during the COVID-19 pandemic, only scratch the surface of this deep-seated issue (22).

These practices do more than merely inflate the cost of healthcare; they fundamentally jeopardize the delivery of essential services and threaten the safety and wellbeing of those involved. Consequently, the quality, accessibility, and integrity of healthcare for Ecuadorian citizens are severely compromised. Related to this, a troubling surge in violence against medical personnel has been witnessed. Last year, Rubén Hernández, the administrator of the “Delfina Torres Hospital” in Esmeraldas province, who was attacked while returning home on March 29 (23). More recently, the murder of Nathaly López, Financial Administrative Director of the “Teodoro Maldonado Hospital” in Guayas province, served as another chilling reminder of the escalating violence (24). These incidences are symptomatic of a more profound crisis and demonstrate the environment of fear in which our health professionals now operate. Many have been compelled to leave their positions, depriving our citizens of their valuable service year (25).

Those of us devoted to public health often find ourselves cornered into expressing our concerns and accusations through academic channels. The prevailing state of fear and violence in Ecuador has led to a reluctance to voice our opinions on social media platforms. This widespread fear is rapidly eroding the quality of our public health system, as many health professionals consider abandoning their posts in favor of the private sector, where they perceive fewer of these problems (26, 27).

Consequently, we find ourselves at a critical juncture where the escalation of these issues threatens to debilitate our healthcare infrastructure. It is imperative for our authorities and the global community to acknowledge this crisis for what it is—a public health emergency. Securing the safety of our healthcare workers, dismantling the mafias operating within the system, and implementing comprehensive, robust measures to prevent corruption are vital.

By confronting these issues head-on, we can aspire to restore integrity in our healthcare system, ensure the safety of our healthcare workers, and enhance healthcare delivery for the Ecuadorian people. Potential solutions include stricter regulatory enforcement, increased transparency in procurement processes, establishment of more effective and participatory monitoring mechanisms, and rigorous prosecution of health-related corruption. Initiatives promoting transparency at all stages of the drug supply chain, especially concerning the quality, availability, and prices of medicines, can also make significant strides in combatting corruption. It is only by taking decisive action now that we can prevent further attrition of our healthcare workforce and safeguard the quality of our public health system.

## Author contributions

EO-P: Conceptualization, Investigation, Methodology, Project administration, Supervision, Visualization, Writing—original draft, Writing—review & editing. JI-C: Formal analysis, Investigation, Methodology, Validation, Writing—original draft, Writing—review & editing. JV-G: Formal analysis, Investigation, Methodology, Validation, Visualization, Writing—original draft.
